# Assembling the components of the quorum sensing pathway in African trypanosomes

**DOI:** 10.1111/mmi.12949

**Published:** 2015-03-04

**Authors:** Binny M Mony, Keith R Matthews

**Affiliations:** Centre for Immunity, Infection and Evolution, Institute for Immunology and Infection Research, School of Biological Sciences, University of EdinburghCharlotte Auerbach Road, Edinburgh, EH9 3FL, UK

## Abstract

African trypanosomes, parasites that cause human sleeping sickness, undergo a density-dependent differentiation in the bloodstream of their mammalian hosts. This process is driven by a released parasite-derived factor that causes parasites to accumulate in G1 and become quiescent. This is accompanied by morphological transformation to ‘stumpy’ forms that are adapted to survival and further development when taken up in the blood meal of tsetse flies, the vector for trypanosomiasis. Although the soluble signal driving differentiation to stumpy forms is unidentified, a recent genome-wide RNAi screen identified many of the intracellular signalling and effector molecules required for the response to this signal. These resemble components of nutritional starvation and quiescence pathways in other eukaryotes, suggesting that parasite development shares similarities with the adaptive quiescence of organisms such as yeasts and *Dictyostelium* in response to nutritional starvation and stress. Here, the trypanosome signalling pathway is discussed in the context of these conserved pathways and the possible contributions of opposing ‘slender retainer’ and ‘stumpy inducer’ arms described. As evolutionarily highly divergent eukaryotes, the organisation and conservation of this developmental pathway can provide insight into the developmental cycle of other protozoan parasites, as well as the adaptive and programmed developmental responses of all eukaryotic cells.

## Introduction

The ability of cells to sense their surroundings and respond to changes in the environment is fundamental to their survival. Monitoring cell density and its regulation is especially beneficial, ensuring the availability of nutrients and space and preventing the accumulation of toxic metabolic waste. This capacity of cells to sense their critical density, referred to as quorum sensing (QS), requires signal molecule/s that serve as reporters of the population density and signal relay components that transmit this information within the cell, causing it to generate a response. The response to increased cell numbers is often a switch to quiescence (usually accompanied by morphogenetic and metabolic changes), a response that promotes survival of the cells under conditions of dwindling resources.

**Figure 1:**
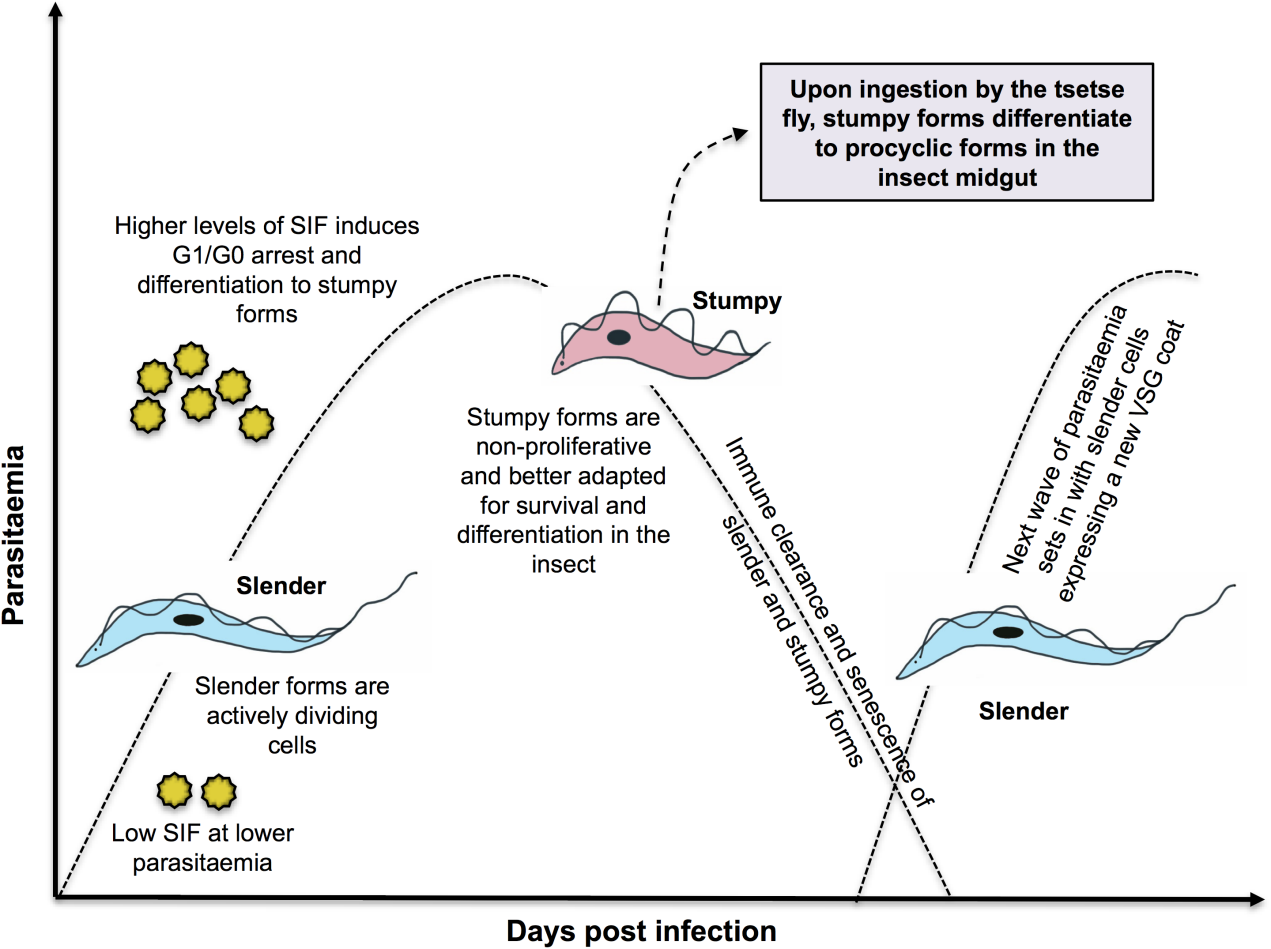
A schematic representation of the infection dynamics of *T. brucei* growth in the mammalian host. Upon reaching a threshold density (and SIF levels) slender forms undergo differentiation to stumpy forms, characterised by G1/G0 arrest as well as morphological changes. Thereafter, the parasitaemia declines, until the next wave emerges due to the proliferation of slender forms with a new VSG coat, this evading antibodies raised to parasites in the first peak. The stumpy forms play a crucial role in transmission, being pre-adapted for survival in the tsetse fly and differentiation to procyclic forms in the tsetse midgut. The growth arrest of stumpy forms is key to maintenance of infection chronicity, as curtailment of the parasitaemia ensures prolonged survival of the host.

African trypanosomes, the causative agents of sleeping sickness in humans and ‘nagana’ in livestock, are protozoan parasites that alternate their lives between a mammalian host and an insect vector, the tsetse fly. In the mammalian bloodstream, the parasites are ‘pleomorphic’ (i.e. of different morphologies) and undergo a developmental transition from rapidly proliferating long ‘slender’ forms to non-dividing short ‘stumpy’ forms, upon attaining a critical cell density (Fig. 1). The slender forms, if prevented from differentiation to stumpy forms, would result in the rapid death of the host through uncontrolled proliferation, as is the case with laboratory-adapted ‘monomorphic’ strains (Turner, 1990). Apart from the visible morphological changes, the differentiation to stumpy forms also entails several physiological changes in the parasite, which promote the survival of stumpy forms during uptake by the tsetse fly, and upon subsequent development to the procyclic forms in the insect midgut. The slender to stumpy switch is thus vital for controlling the parasitaemia as well as for efficient transmission of the parasite to the fly, both of which aid in the successful maintenance and spread of infection.

## The differentiation signal

Pleomorphs reportedly need to attain a threshold parasitaemia (apparently variable in different hosts) before differentiation and proliferation arrest in G1/G0 as stumpy forms can occur (Seed and Sechelski, 1989). Similarly, the cell cycle arrest of pleomorphic parasites grown *in vitro* is linked to their cell density, and this is irrespective of their initial seeding density (Reuner *et al*., 1997). These observations suggested the existence of a density sensing mechanism in trypanosomes and, further, the ability of plasma from infected animals at peak parasitaemia to inhibit trypanosome proliferation proposed the involvement of a secreted factor (Seed and Sechelski, 1989), although the accumulation of toxic inhibitors was not ruled out.

This scenario of trypanosomes undergoing growth arrest upon reaching a threshold density is resonant of QS pathways, widely described in bacteria and several lower eukaryotes. Gram-negative bacteria largely employ N-acyl-homoserine lactones (AHLs) as auto inducers of QS signalling cascades. The AHLs are freely diffusible molecules that can bind to and activate a transcriptional activator, which in turn induces expression of target genes. Another class of molecules that mediate similar effects is the 4-quinolones. On the other hand, Gram-positive bacteria use small post-translationally processed peptides as signal molecules. These peptides have been shown to interact with a histidine kinase two-component signal transduction system (Gonzalez and Keshavan, 2006; Atkinson and Williams, 2009; Deep *et al*., 2011). Beyond bacteria, several eukaryotes such as yeast and other fungal species communicate through diffusible molecules including farnesol and tyrosol and other small molecules (Hogan, 2006). Similarly, the social amoeba, *Dictyostelium discoideum*, in response to starvation, secretes an array of factors, including the glycoprotein conditioned medium factor. Additionally, the cells secrete pre-starvation factors during growth, which accumulate in the medium and hence serve as indicators of cell density. Other extracellular signals such as a chlorinated hydrocarbon called differentiation-inducing factor, the steroid, GABA and small peptides have also been shown to mediate QS in this slime mold (Clarke and Gomer, 1995; Gomer *et al*., 2011).

In trypanosomes, while identification of the QS signalling molecule has evaded us to date, the evidence is that pleomorphs (and monomorphs) secrete a soluble, low molecular weight, heat stable factor/s termed stumpy induction factor (SIF). SIF is of parasite origin, and its accumulation in the culture medium/blood stream, with increasing parasite density, is proposed to act as the trigger for the slender to stumpy differentiation. This factor is believed to act on the same population that produces it, such that it induces an autocrine response. Monomorphs, in contrast, seem to be ‘signal-blind’ to the factor they produce, thus providing them with a growth advantage *in vitro* (Vassella *et al*., 1997). This selective advantage is restricted to *in vitro* growth; *in vivo*, host longevity and the need for tsetse transmission would represent an important counter-selection to retain pleomorphism. This probably explains why monomorphs are not favoured in nature and reinforces the importance of density-dependent growth in the success of the trypanosomes' proficient parasitic lifestyle (MacGregor *et al*., 2012).

## The SIF signalling pathway

Lack of information on the chemical identity of SIF has made it difficult to understand the production of the signal, its turnover and the molecular cascade involved in relaying this signal. Early studies on slender forms noted a two- to threefold increase in cyclic AMP (cAMP) levels at peak parasitaemia followed by a decline in its levels during the transition to stumpy forms (Mancini and Patton, 1981). Consistent with that, addition of conditioned medium containing SIF also resulted in a similar elevation in cAMP levels in pleomorphs, indicating a potential role for this secondary messenger in QS signalling (Vassella *et al*., 1997). A SIF-like growth inhibitory effect was also seen when pleomorphs were exposed to the cell permeable cAMP analogue (8-pCPT-cAMP) further supporting its possible role in this pathway (Vassella *et al*., 1997). Nevertheless, it was later shown that the active modulator was actually the hydrolysis product of 8-pCPT-cAMP, namely 8-pCPT-AMP (or its adenosine equivalent), as non-hydrolysable versions of 8-pCPT-cAMP were incapable of inducing growth arrest (Laxman *et al*., 2006). Consequently, this ruled out the participation of a canonical cAMP signalling mechanism in the differentiation process. Moreover, in contrast to their differential responsiveness to SIF, both pleomorphs and monomorphs responded to 8-pCPT-cAMP demonstrating that monomorphs, though blind to SIF, still probably retained some of the components of the signalling pathway that drives stumpy formation (Laxman *et al*., 2006).

**Figure 2:**
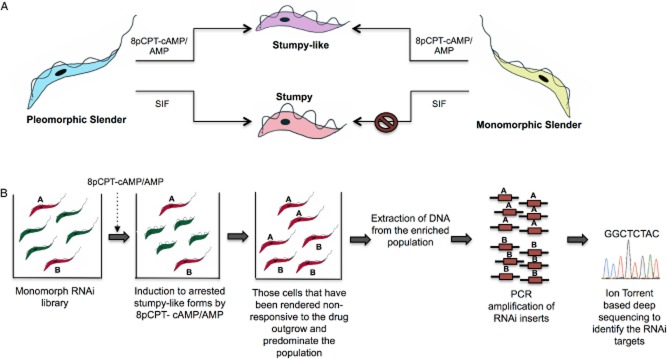
*In vitro* induction of stumpy-like forms using 8pCPT-cAMP/AMP (A) and use of this response in a genome-wide RNAi screen to identify components of the stumpy induction pathway (B).A. Pleomorphic parasites are capable of responding to SIF, giving rise to stumpy forms. However, monomorphs are non-responsive to SIF but, instead, are capable of being induced to stumpy-like forms by cell permeable, hydrolysable, cAMP/AMP analogues.B. A monomorphic RNAi library was exposed to 8pCPT cAMP/AMP. Those parasites that had a gene required in the cAMP/AMP response pathway depleted (red), remained slender and continued proliferating, whereas the others (green) underwent growth arrest. The resistant parasites eventually outgrew and predominated the population. DNA was extracted from the selected cells, the RNAi inserts amplified by PCR and then subjected to ion-torrent deep sequencing to identify the genes (A and B) involved in relaying the 8pCPT-cAMP/AMP signal. The identified genes were then validated through individual RNAi lines in pleomorphs to confirm their role in physiological SIF signalling (adapted from Mony *et al*., 2013).

This ability of monomorphs to respond to 8-pCPT-cAMP/AMP and undergo growth arrest has been exploited in a genome-wide RNAi library screen that sought to select parasites that were unresponsive to 8-pCPT-cAMP/AMP after gene knockdown. The monomorphic library, capable of tetracycline-induced gene silencing on a genome-wide scale, was exposed to 8-pCPT-cAMP/AMP, following which, growth was monitored and compared with the uninduced set. The parasites that failed to undergo growth arrest were believed to continue proliferation due to the knockdown of a gene required for sensing the 8-pCPT-cAMP/AMP signal. Deep sequencing of the RNAi inserts enriched in the selected 8-pCPT-cAMP/AMP resistant parasite population divulged a collection of genes representing various steps of a typical signalling pathway and likely to be involved in the 8-pCPT-cAMP/AMP response (Fig. 2). Crucially, when many of these genes were individually knocked down in pleomorphs by RNAi, they conferred resistance to SIF *in vivo* thereby confirming their involvement in the biologically relevant QS signalling pathway. This demonstrated that the 8-pCPT-cAMP/AMP–mediated pathway intersected with the SIF signalling pathway at least to some extent, though inevitably molecules involved in the reception of the SIF signal were missing given the use of a cell permeable signal in the screen (Mony *et al*., 2013).

This extensive screen thus gave the first insight into the complex machinery responsible for the slender to stumpy transition. The identified components ranged from characteristic cell signalling molecules such as phosphatases and kinases, to post-transcriptional gene regulators such as RNA binding proteins. Although arranging these components in precise order in the signalling cascade represents a significant experimental challenge, it is noteworthy that the list of genes identified overlap with those involved in nutritional stress induced dormancy of yeast and other lower eukaryotic cells (Broach, 2012; De Virgilio, 2012). It is tempting, therefore, to draw parallels with eukaryotic cell quiescence pathways to facilitate construction of a framework for the QS pathway in trypanosomes.

In the following sections we detail several of the protein families implicated in the control of stumpy formation and relate this to what is known in other eukaryotic organsims.

### A. Signalling molecules

#### Phosphatase cascades in trypanosome differentiation

Protein phosphorylation plays a major role in the control and regulation of cellular functions in eukaryotic cells. This post-translational modification occurs mainly on serine (Ser) or threonine (Thr) residues, making Ser/Thr protein phosphatases (PPs) key players in the eukaryotic cell. The Ser/Thr protein phosphatases of type 1 (PP1) are the most abundant and among the best characterised protein phosphatase family. The PP1 catalytic subunit can interact with a large number of regulatory proteins, resulting in a conformational change of the phosphatase and a specific dephosphorylation of the target. Protein tyrosine phosphatases (PTPs), which also include dual-specificity phosphatases, dephosphorylate proteins at the tyrosine residues and play a major role in signal transduction. The ability of phosphatases to interact with multiple substrates explains their involvement in a wide range of cellular activities although there is limited information on their role in trypanosome biology. Nonetheless, a tyrosine phosphatase in *T. brucei*, *Tb*PTP1, has previously been shown to play a key role in differentiation of the stumpy forms to procyclic forms (Szoor *et al*., 2006) with this acting on a serine threonine phosphatase that is targeted to glycosomes (Szoor *et al*., 2010). For the differentiation of slender parasites to stumpy forms, a PP1 and a dual specificity phosphatase (DsPhos) have been implicated experimentally (Mony *et al*., 2013).

In yeast cells, PP1 (Glc7) plays a vital role in glucose signalling by interacting with a large number of regulatory proteins (e.g. Reg1). Glc7 is then capable of inactivating 5'AMP-activated protein kinase (AMPK) (Snf1 in yeast), which in turn transmits the glucose starvation signal (Sanz *et al*., 2000). Glc7 also dephosphorylates Msn2 and Msn4, the major stress-responsive transcription factors, causing their re-localisation from the cytoplasm to the nucleus and hence activation of stress-responsive genes (Gorner *et al*., 1998; 2002[Bibr b27],[Bibr b28]). In trypanosomes, members of a PP1 array [Tb927.4.3620–3640; PP1-3,4,5 (nomenclature based on Li *et al*., 2006)] were shown to be key inducers of stumpy formation, with their simultaneous RNAi-mediated knockdown preventing differentiation (Mony *et al*., 2013). Conversely, overexpression of one of these PP1 members drives cells into G1 arrest (Mony and Matthews, unpubl. obs.). The coincident identification of an AMPK as a stumpy inducer, in the same RNAi screen, hints at the possible functioning of a similar mechanism in *T. brucei*. For example, one could speculate that PP1 acts upstream of AMPK in the SIF pathway such that AMPK depletion by RNAi might prevent the cell-cycle arrest generated upon PP1 overexpression, although PP1 might also have other targets in the pathway. Dual specificity phosphatases (DsPhos) in yeast have also been shown to regulate cell growth via regulation of the protein kinase A (PKA) cascade (Beeser and Cooper, 2000) and could function analogously in trypanosomes, given that both a predicted DsPhos (Tb927.7.7160) and a PKA regulatory subunit (Tb927.11.4610) were identified in the screen for stumpy formation. Apart from these known functions, the phosphatases might also be operating through the deactivation of stumpy inhibitors or the slender ‘retainers’ (explained in a later section), molecules that would not have been identified in the genome-wide RNAi screen for drivers of stumpy formation.

#### Kinase-mediated signalling of nutrient starvation

A kinome-wide RNAi screen in *T. brucei* recently identified several novel kinases that functioned as cell cycle regulators as well as two kinases, RDK1 and RDK2, that apparently played a role in differentiation to procyclic forms (Jones *et al*., 2014). Two of the signalling cascades central to cellular quiescence in eukaryotes are those involving PKA and the target of rapamycin (TOR). The PKA pathway in yeast is largely responsible for the transcriptional changes associated with changes in glucose availability and leading to cellular quiescence (Zaman *et al*., 2008). The binding of cAMP to the regulatory subunit (PKA-R) alleviates the repression of the catalytic subunit (PKA-C), which in turn can now phosphorylate its substrates resulting in enhanced cellular proliferation. Thus, the increase in intracellular cAMP levels encountered during glucose rich conditions activate PKA-C and ensure continued cell proliferation. Conversely, glucose depletion is followed by a decrease in cAMP levels leaving PKA-R free to bind to PKA-C, leading to its inhibition and hence a G1 arrest (Griffioen *et al*., 2000).

Trypanosomes have a single gene encoding PKA-R and three orthologues for PKA-C, with the PKA-R gene (Tb927.11.4610) being identified in the RNAi screen for stumpy inducers. The RNAi-mediated ablation of PKA-R (found in the flagellar matrix) in monomorphs inhibited the motility of the parasites (Oberholzer *et al*., 2011), a phenomena thought to be regulated by cyclic nucleotide signalling as well. In *D. discoideum*, disruption of PKA-R is known to keep PKA-C in a constitutively active state (Simon *et al*., 1992) such that in trypanosomes, the knockdown of PKA-R might keep PKA-C constitutively active and hence in a proliferative slender state. However, knockdown of PKA-R via RNAi in pleomorphs proved lethal for the parasite, probably due to a possible role in other cellular functions (as mentioned above) such that its rapid experimental validation for a role in stumpy induction (SI) was prevented (Mony *et al*., 2013).

Parallel to the PKA pathway is the YAK kinase pathway that acts as a suppressor of proliferation (Garrett *et al*., 1991). In budding yeast, the YAK homologue, Yak1p has a function dependent on its cellular localisation. Upon glucose starvation, it becomes translocated to the nucleus and more highly expressed and is believed to phosphorylate PKA-R (Griffioen *et al*., 2001) along with other substrates. Phosphorylation of PKA-R in the nucleus results in its relocation to the cytoplasm, which probably results in inactivation of the PKA pathway and ultimately reduced cell proliferation (Zaman *et al*., 2008). The homologue of YAK in *Dictyostelium*, *YAKA*, induces a similar growth arrest (Souza *et al*., 1998), whereas in fission yeast, a related dual specificity Yak-related kinase, Pom1p, generates a morphogenic gradient that prevents mitotic progression until cells reach an appropriate length (Bahler and Pringle, 1998; Aranda *et al*., 2011).The YAK kinase gene in *T. brucei* (Tb927.10.15020) has a potentially comparable function in cell cycle inhibition, as its depletion via RNAi results in prolonged cell proliferation and maintenance of slender forms (Mony *et al*., 2013) through as yet uncharacterised pathways. However, whether relocalisation to the nucleus contributes to its regulation of proliferation is unclear, given the focus of trypanosome gene regulation on post-transcriptional rather than transcriptional events.

Members of the AMPK family (Snf in yeast and plants) act as major regulators of cellular energy homeostasis in eukaryotic cells. The AMPK cascade is regulated by the AMP:ATP ratio and hence acts as an energy sensor. A decline in ATP levels, along with a concomitant rise in AMP, activates this kinase, which then phosphorylates a range of downstream targets to bring about changes that reduce catabolic processes within the cell. AMPKs are thus capable of responding to a reduction in ATP levels (that may be brought about by nutrient depletion) by pushing the cell towards a state of reduced activity, in order to maintain the AMP:ATP ratio. Interestingly, AMPK is also known to be an inhibitor of the mTOR pathway (Carling *et al*., 2011). In trypanosomes, TOR4 was shown to be a negative regulator of SI (Barquilla *et al*., 2012), demonstrated by its knockdown in monomorphs, which drove the cells to develop features characteristic of stumpy forms. By this logic, one would thus expect that AMPK knockdown would maintain TOR4 in its active state and hence prevent differentiation, a prediction supported by the identification of an AMPK catalytic subunit in the RNAi screen for SI (Mony *et al*., 2013).

The mitogen-activated protein kinase (MAPK) cascade in yeast is involved in stress responses as well as cell cycle regulation. This cascade, which involves a module of three protein kinases (MAPKKK, MAPKK and MAPK) that sequentially activate one another by phosphorylation, form a crucial part of intracellular signalling pathways in eukaryotic cells (Gustin *et al*., 1998). The most upstream kinase of this cascade is MAPKKK (MEK), and in trypanosomes, Tb927.2.2720 encodes a protein that contains the MEK domain. Knockdown of this gene renders pleomorphs non-responsive to SIF, suggesting a role for the MAPK pathway in QS signal transduction in trypanosomes (L. McDonald and K. R. Matthews, unpubl. obs.). Because in other eukaryotes, these kinases play a key role in conveying extracellular signals, it is likely that in trypanosomes, Tb927.2.2720 acts closely downstream of the unidentified SIF receptor, transmitting the external cell density signals to the cellular targets. This would thus place the MEK quite high up in the QS signalling cascade.

The never in mitosis A (NIMA)–related kinases or NEKs, a class of serine/threonine kinases, were originally identified in a genetic screen for cell division cycle mutants in *Aspergillus nidulans*. The role of NEKs in cell cycle check point control has been extensively reviewed in (Moniz *et al*., 2011; Fry *et al*., 2012) and may play a similar role in trypanosomes, where the NEK kinase family is considerably expanded (Jones *et al*., 2014). Members of this family are also implicated in QS as RNAi of one NEK family (Tb927.10.5930/5940/5950; these being indistinguishable by RNAi) delayed differentiation to stumpy forms, although this phenotype was less stringent than, for example, ablation of PP1 (where stumpy formation was completely abolished). This might reflect incomplete gene silencing or an incomplete penetrance of the genetic effect.

In summary, both protein phosphatases and protein kinases have been identified as regulators of the transition between slender and stumpy forms, though the targets upon which they operate are unknown. Nonetheless, phosphorylation is an expected contributor to the regulation of the signalling molecules themselves and also the effector molecules for differentiation upon which they may act, such as RNA binding proteins. Phosphoproteomic datasets analysing cultured bloodstream and procyclic forms (Nett *et al*., 2009; Urbaniak *et al*., 2013) have already highlighted the potential for phosphorylation of many of the components identified from the screen for stumpy inducers; the consequences of these specific post-translational modifications will require individual experimental analysis.

### B. Gene regulators

The ultimate modulators in any signalling cascade are those that bring about gene expression and translational changes. Although the transcriptional silencer Imitation SWItch (ISWI) (Hughes *et al*., 2007; Stanne *et al*., 2011) was detected in the screen for stumpy inducers, more focus has been placed on post-transcriptional regulation because several studies have found RNA-binding proteins (RBPs) to play a key role in various stages of the trypanosome life cycle (Clayton, 2014; Kolev *et al*., 2014). Although RBPs have been found to function in the bloodstream to procyclic transition and in metacyclogeneis (Kolev *et al*., 2012), RBP7 [representing two almost identical genes: RBP7A (Tb927.10.12090) and RBP7B (Tb927.10.12100)] is the sole candidate that has been experimentally demonstrated to be involved in the slender to stumpy differentiation. This protein has a single RNA recognition motif (RRM) and, although its overexpression prematurely drives parasites to stumpy forms, its knockdown results in a delayed response to SIF. Analysis of the transcripts altered in abundance upon perturbed RBP7 expression (i.e. via RNAi or RBP7B overexpression) revealed, for example, the upregulation of several histone transcripts upon RBP7 knockdown, probably reflecting the continued proliferation of parasites as slender forms. In contrast, RBP7 overexpression increased the abundance of mRNAs of several other RNA binding proteins, but also certain procyclic form associated transcripts, which could reflect preparation for the next stage in the life cycle once stumpy cells are taken up by the tse-tse fly. The RRM motif found in RBP7 has sequence homology (∼ 47%) to the RNA binding motif of the FCA protein in plants that is involved in the developmental transition to flowering (Macknight *et al*., 1997).

As well as the proteins containing identifiable RNA binding motifs, a recent genome wide screen to identify posttranscriptional regulators in *T. brucei* (Erben *et al*., 2014) revealed three hypothetical proteins that had already been identified as regulators of stumpy formation (Mony *et al*., 2013) namely, Tb927.11.6600 (HYP 1), Tb927.9.4080 (HYP 2) and Tb927.11.2250 (HYP 12). Of these, HYP1 downregulates mRNAs when artificially tethered to transcripts, whereas HYP2 and HYP11 upregulated bound mRNAs. HYP 1 and 2 were shown to be involved in SI through RNAi-mediated knockdown in pleomorphs (Mony *et al*., 2013) and possibly drive stumpy formation by destabilising slender form ‘retainers’ or inhibitors of stumpy forms (for HYP1) or stabilising drivers of stumpy formation (HYP2). HYP12, in contrast, has not yet been validated to have a role in stumpy formation through the creation of individual RNAi lines, but has nevertheless, been shown to bind RNA (Erben *et al*., 2014).

### C. Hypothetical proteins

Elucidating the exact role of a protein annotated as ‘hypothetical’ is a challenging task in trypanosome biology due to the lack of any information on its probable function. However, the fact that many hypothetical proteins identified in the screen are conserved across kinetoplastid species as well as the demonstration that two of them (HYP1 and HYP2) drive stumpy formation suggests they are fundamental components of a conserved cell communication or cell quiescence pathway found in other kinetoplastids. One observation from Table 1 (a list of hypothetical genes driving stumpy formation) is that some of the hypothetical proteins harbour domains predicted to be involved in preprotein import into mitochondria (Tb927.4.3650 (HYP 4) and Tb927.9.13530 (HYP 6)) as well as there being a mitochondrial small subunit (SSU) ribosomal protein Tb927.11.11470 (HYP 13). This could mean that mitochondrial proteins contribute to stumpy formation and are not simply reflective of the mitochondrial elaboration that characterises stumpy formation. Also noteworthy is the identification of proteins with probable functions in ubiquitination (i.e. Tb927.8.2860, HYP 5; and Tb927.2.4020, a ubiquitin activating enzyme). These genes may be involved in the degradation of the slender cell proteins, which are no longer required in the next developmental stage or that act as repressors of stumpy formation. These predictions not only suggest that stumpy formation might be a multifaceted process, but also highlight the importance of analysing these hypothetical proteins in uncovering aspects unique to kinetoplastid QS.

**Table 1:** Predicted functions of hypothetical proteins identified in the genome wide RNAi screen for stumpy inducers

Gene Id	Name	Domains	Predicted function
Tb927.11.6600	Hyp 1	No conserved domain	Downregulates artificially tethered transcripts (Erben *et al*., 2014)
Tb927.9.4080	Hyp 2	Truncated AAA+ and Adenylation domains	ATP-dependent polynucleotide ligase; upregulates artificially tethered transcripts (Erben *et al*., 2014)
Tb927.4.670	Hyp 3	GAF domain	Small molecule (cyclic nucelotide) binding, protein–protein interactions; Found in phosphodiesterases (Heikaus *et al*., 2009)
Tb927.4.3650^a^	Hyp 4	No conserved domains	May function as a mitochondrial import receptor subunit TOM6 homolog (34%) involved in translocation of preproteins across mitochondrial outer membranes (Dukanovic *et al*., 2009)
Tb927.8.2860	Hyp 5	Truncated Mod (r) domain	Endosomal protein sortin; recognition of monoubiquitinated cargo proteins, mainly surface proteins such as transporters and receptors (Winter and Hauser, 2006)
Tb927.9.13530	Hyp 6	Pam16	Preprotein import into the mitochondrial matrix (Frazier *et al*., 2004)
Tb927.10.12110^a^, ^b^	Hyp 7	No conserved domain	Membrane-associated guanylate kinase, WW and PDZ domain-containing protein 1 (48%)
Tb927.11.300	Hyp 8	Truncated Bud13 domain	Pre-mRNA splicing and retention
Tb927.11.750	Hyp 9	Domains of Helicase, RING-finger, zf-PARP; disrupted domain of DEAD-like helicase	Transcription/DNA replication, recombination, and repair; RING-finger: DNA binding, cell signalling, ubiquitination (Tuo *et al*., 2012)
Tb927.11.1640	Hyp 10	No conserved domains	None
Tb927.11.2250	Hyp 11	Truncated domain of spumavirus Gag protein	Genome packaging, virion assembly, trafficking and membrane targeting in foamy viruses (Goldstone *et al*., 2013); upregulates artificially tethered transcripts (Erben *et al*., 2014)
Tb927.11.6610^b^	Hyp 12	Truncated RRM domain	Regulation of post-transcriptional gene expression
Tb927.11.11470	Hyp 13	No conserved domains	Putative mitochondrial SSU ribosomal protein

a Function prediction based on sequence homology rather than presence of conserved domain, with % identity in parentheses.

b The library RNAi fragment spanned more than one gene; hence this gene was not unambiguously identified.

Except Hyp1 and Hyp2, none of the other hits have been experimentally validated.

### D. Expression site (ES) components

Pleomorphic parasites in the mammalian blood stream follow a characteristic undulating growth pattern, at least in experimental infections, with slender forms dominating until close to peak parasitaemia. These are replaced by transitional intermediate forms and then by the stumpy forms that predominate the declining phase until the next upsurge of slender forms sets in (Fig. 1). The slender forms that emerge in each new wave have an antigenically distinct coat of variant surface glycoprotein (VSG), allowing them to proliferate in the face of host immunity to parasites generated in the first wave, such that antigenic waves and the slender to stumpy differentiation are temporally connected. However, recent evidence has highlighted the possibility of a mechanistic linkage also. The parasite harbours several hundred VSG genes clustered at subtelomeric regions, plus about 15–20 ESs of which only a single ES is active at any given time (Pays *et al*., 2001). Each of these ESs also contains several other genes known as expression site-associated genes (ESAGs). Although ES control and differentiation were believed to be mostly independent, there is some evidence for co-ordination. Firstly, the slender to stumpy differentiation has been demonstrated to be accompanied by the silencing of the active ES (Amiguet-Vercher *et al*., 2004), though the actual molecular trigger is not known. Also, VSG synthesis and cell cycle progression are closely linked as RNAi-mediated knockdown of VSG resulted in a reversible cell cycle arrest, albeit at pre-cytokinesis rather than G1 (Sheader *et al*., 2005; Smith *et al*., 2009). Most recently, Batram *et al*. (Batram *et al*., 2014) have shown that ES attenuation, and more specifically depletion of three ESAGs (ESAG 1, 2 and 8), could trigger cell cycle arrest and the expression of the stumpy specific marker PAD1 (Dean *et al*., 2009). However, unlike SIF-mediated differentiation, this ES attenuation-mediated G1 arrest was reversible. It is thus possible that the G1 block observed after ES attenuation might be a consequence of the lack of the ESAG products. Although ESAG1 function is poorly understood, this protein is suggested to be localised to the cell surface (Cully *et al*., 1986; Pays *et al*., 2001). The function of ESAG2 is also unknown, but the presence of hydrophobic stretches in its encoded protein sequence indicate that this maybe a surface protein as well. ESAG 1 and 2 have also been localised to the flagellar pocket and could function as surface receptors (Pays *et al*., 2001). ESAG8, in contrast, has a nucleolar as well as cytoplasmic localisation and interacts with a Pumilio domain containing protein, Puf1, denoting that it could act as a transcriptional or translational regulator (Hoek *et al*., 2002). In consequence, one could envision that these ESAGs are involved in the proliferative physiology of the slender forms such that their depletion might generate a nutritional starvation or stress-like environment that invokes a stumpy formation response through a signalling pathway that intersects with components of the SIF pathway. Although this could be beneficial to the cell by ensuring that successful ES switching is achieved (accompanied by ESAG1, 2 and 8 re-expression) and proliferation can recommence, the frequency of switching in the bloodstream parasite population would render this a minor contributor to the overall development of arrested, transmissible, stages in the blood.

## Integrating the known networks

Although the discovery of various components of the SI pathway by the genome-wide screen has given a substantially better understanding of how trypanosomes positively drive stumpy formation, earlier studies had already identified possible negative regulators of the process. For example, the ablation of a zinc finger kinase (ZFK) in pleomorphs inhibited growth and also increased the rate of slender to stumpy transition *in vitro*, though not *in vivo* (Vassella *et al*., 2001). Similarly, the creation of a null mutant for a MAPK homologue, TbMAPK5, gave a similar phenotype with the exception that the phenotype was also seen *in vivo*, thus reducing the chronicity of mouse infections (Domenicali Pfister *et al*., 2006). Although the ZFK and MAPK knockouts could induce differentiation only in pleomorphs, RNAi of the TOR kinase, TbTOR4 in monomorphs triggered a transition to the stumpy form (Barquilla *et al*., 2012), as highlighted earlier. This may reflect the existence of redundant signalling pathways or a defect in the pathway in monomorphs at a point downstream of ZFK and MAPK but upstream of TbTOR4.

**Figure 3:**
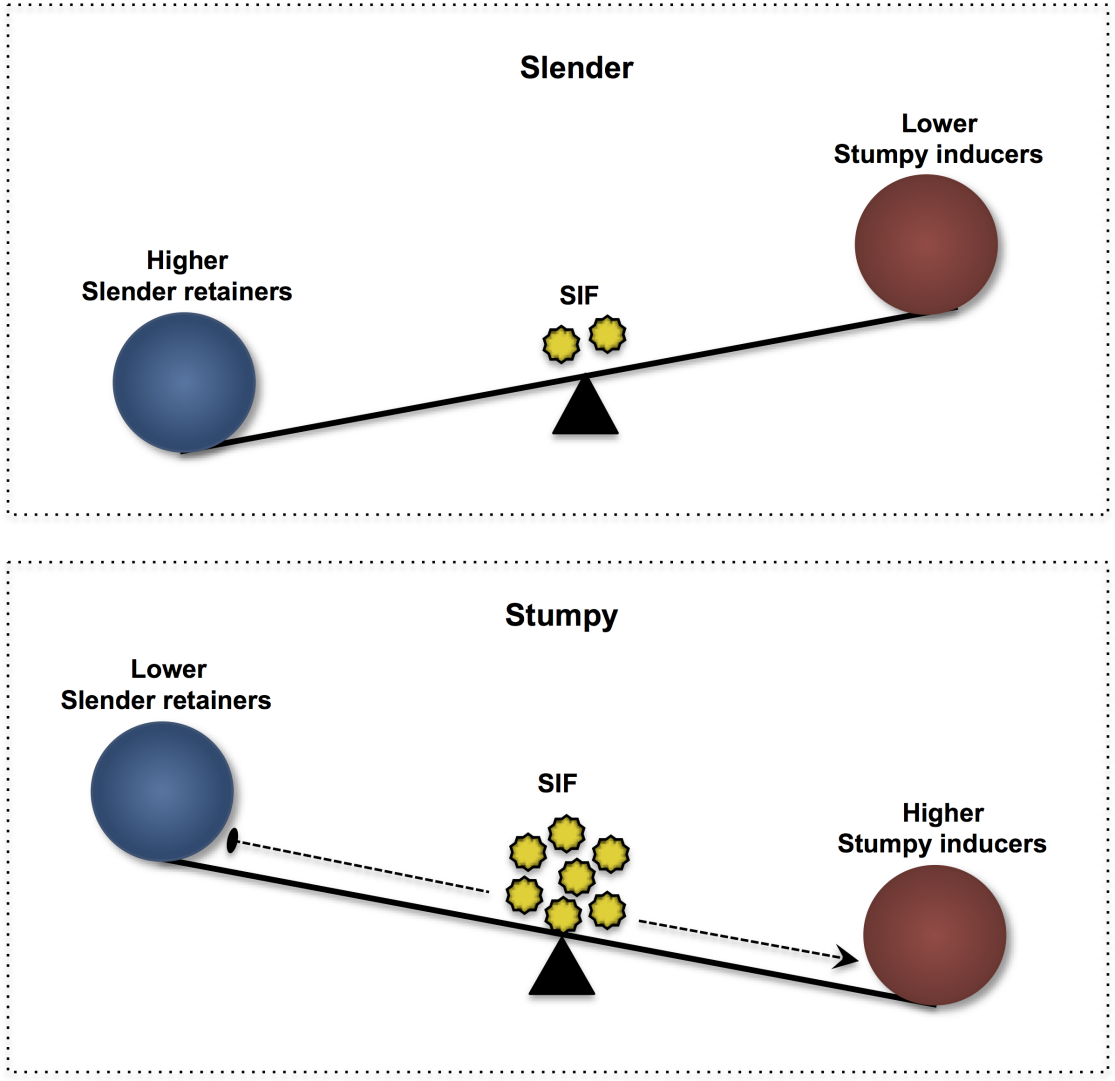
The balance between ‘slender retainers’ (SR) and ‘stumpy inducers’ (SI) controls stumpy formation. The slender cells remain proliferating as long as the levels of SR are high and SI are low. However, upon an increase in cell density, SIF accumulates, triggering a quorum sensing like response that induces the activation of SIs with a concomitant repression of the SRs. It is the combined action of ‘switching-off’ of the slender retention (SR) arm and ‘switching-on’ of the stumpy induction (SI) arm that ultimately drives the formation of stumpy cells.

Combined, one can speculate that trypanosomes have a dual mechanism for controlling differentiation, comprising of a slender retention (SR) as well a SI arm (Fig. 3). A set of genes, the ‘slender retainers’ (ZFK, MAPK5, TOR4, etc.) is constitutively expressed, and their products keep the cell in an actively proliferating state. This slender state is retained until SIF accumulation reaches threshold levels, triggering the ‘nutritional stress-like’ response, which leads to concomitant repression of the ‘slender retainers’ and activation of ‘stumpy inducers’. This tip in the equilibrium towards SI, apart from causing cell cycle arrest, also starts preparing the cell for its next life cycle stage in the insect by re-activating mitochondrial functions required for oxidative phosphorylation as well as by removing the proteins that were once needed for the maintenance of slender forms (for example by ubiquitination). Thus, the transformation to stumpy forms would be rendered irreversible through the coupled inactivation of the SRs and activation of SIs. Hence, it is the fine control of these two processes that may determine the balance of proliferation versus differentiation, though the relative dominance of SR versus SI components remains to be determined, as does the point of commitment in this transition. Another aspect worth noting is the possibility of redundancy in the QS sensing pathway, as seen in the case of the response to nutritional starvation in yeast (Granek *et al*., 2011).

A less investigated but crucial aspect of the differentiation process is the timing of the onset of the commitment to differentiation from the long, slender to the short, stumpy form. Although asymmetric division was suggested to be the means by which trypanosomes underwent commitment and morphological transformation (Tyler *et al*., 2001; Sharma *et al*., 2008), it would be difficult, with currently available data, to map out the exact timeline of events comprising the response to SIF and the subsequent cell cycle arrest, although the subsequent organelle reorganisation and changes in cell shape have been described (Vanhollebeke *et al*., 2010; MacGregor *et al*., 2012). However, one could speculate that SIF induced downregulation of SRs could be augmented by the dilution of these factors during the division of slender cells undergoing commitment to differentiation but that have not yet undergone arrest (MacGregor *et al*., 2011). Any asymmetry in these divisions (and the distribution of SRs) could determine whether one or both daughters ultimately commits to arrest and differentiation.

One piece of experimental evidence that supports a tension between stumpy inhibition and induction is the observation that, despite effective mRNA and protein elevation, the overexpression of ectopic RBP7B does not drive complete stumpy formation (Mony *et al*., 2013). Although other reasons could contribute to inefficient differentiation (such as the requirement for an interacting protein or a post translational modification of RBP7 for it to be completely active), this phenotype could also be due to the continued presence of ‘slender retainers’. Also possible is the presence of multiple or hierarchical ‘stumpy inducing’ arms in the pathway such that cell cycle arrest might precede (and be dissociable from) irreversible commitment and morphological transformation to stumpy forms, a concept supported by the observed effect of ESAG depletion on reversible G1 arrest and the fact that AMPK and TOR kinase have antagonising consequences (see above) on stumpy formation. Clearly, it would be enormously valuable to identify slender retainers to facilitate a better understanding of the molecules that oppose the action of the stumpy inducers identified in the genome-wide RNAi screen and the QS pathway. The availability of an over-expression library (Erben *et al*., 2014) now makes it feasible to screen for such negative regulators of stumpy formation.

## QS interference, interspecies interactions and communication with the host

Studies on QS in prokaryotes have revealed that the communication between cells can be far more complex than a simple autocrine effect. Apart from communicating with themselves, bacteria have been shown to intercept the signals of others in a niche containing several species (Deng *et al*., 2014). Numerous eukaryotes have also been shown to be capable of perceiving prokaryotic QS signals (Atkinson and Williams, 2009; Deng *et al*., 2014). Although such information on QS in trypanosomes is currently lacking, it represents an area of interest because trypanosomes not only interact within and with the host but also exist in the mammalian bloodstream in the company of other parasites. Infections with multiple species of trypanosomes are common in sub-Saharan Africa (Cox *et al*., 2010), and there are currently uncharacterised possible interactions between them, involving either competition or co-operation. Moreover, the difficulty in producing biochemically and morphologically replete stumpy forms *in vitro* also suggests that the host bloodstream environment could contribute additional physical or biological factors in the process. Understanding these *in vivo* contributors will be important in dissecting the stumpy formation pathway and its impact, in different hosts, on parasite virulence and transmission. Of course, a detailed knowledge of the molecular components of the SIF pathway could also prove useful in the development of drugs that interfere with or mimic parasite communication, thus perturbing parasite virulence and transmission potential.

## Conclusions

**Figure 4:**
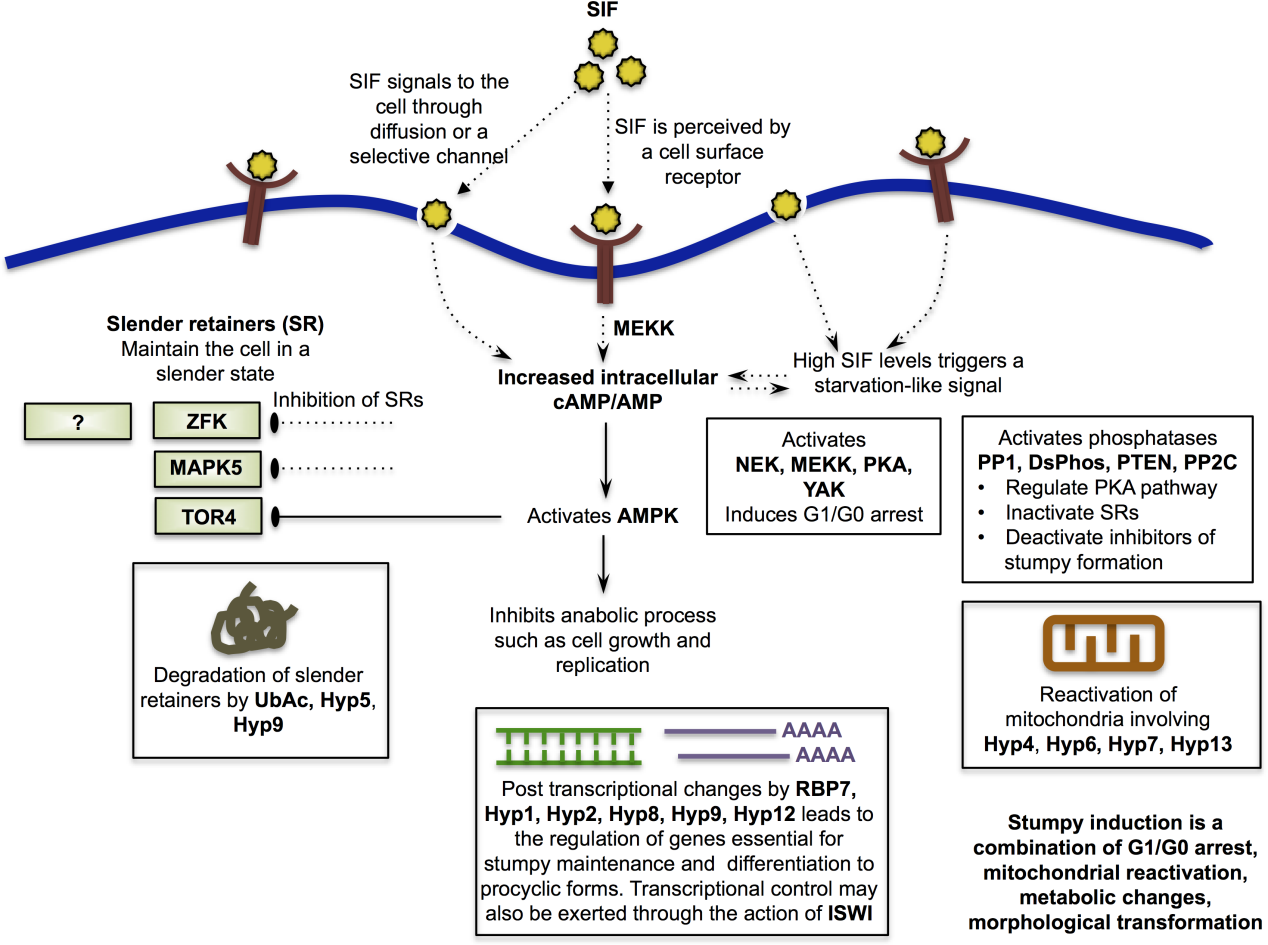
A hypothetical framework for the molecular control of stumpy formation. The figure shows an integration of all known networks linked to starvation responses and genes identified in the genome-wide RNAi screen for drivers of stumpy formation. Many of the components included remain to be validated in independent RNAi or knockout lines and so their inclusion is speculative.

With the development of tools such as the genome-wide RNAi (Alsford *et al*., 2011) and overexpression (Erben *et al*., 2014) libraries, it has now become possible to carry out selective screens in *T. brucei* to identify the molecular components of biological pathways in addition to simple drug resistance mechanisms. One such screen, using a chemical inducer (8-cPT-cAMP/AMP) of stumpy-like forms *in vitro* has led to the discovery of various components of the SIF pathway (Mony *et al*., 2013). Although this review has attempted to assemble those components based on *in silico* information, the existing literature as well as experimental data from other eukaryotes (Fig. 4), validation of these hypotheses through experimentation is a significant challenge, especially where pathway components have other roles in the cell's physiology. Indeed, the identified pathway components are very likely to be part of an incomplete list, as stumpy inducers that are also essential proteins in the parasite would not have become enriched in the screen. It is thus crucial to dissect and further add to this pathway through multiple approaches. As an example, a recent high throughput chemical screen identified a novel chemical inducer of stumpy forms (MacGregor *et al*., 2014) allowing a chemical-genetic dissection of the pathway. Phosphoproteomic and transcriptomic approaches will also help in the identification of potential targets of the known components of the SIF pathway, especially of the phosphatases and kinases. Nonetheless, although a great deal of further effort is needed to characterise this pathway in detail, it already represents the most comprehensively characterised signalling pathway in any eukaryotic parasite. Hence, discoveries related to trypanosome development could inform similar cell–cell communication pathways in other parasites such as that regulating gametocytogeneisis in *Plasmodium* (Ikadai *et al*., 2013) as well as the regulation of developmental responses of other kinetoplastids, including *T. cruzi* and *Leishmania spp*. The evolutionarily ancient position of trypanosomes in the eukaryotic lineage also has the potential to inform understanding of the molecular basis of cellular quiescence in other cells including those of mammals, whose breakdown is a key contributor to the development of many cancers.
